# Adipokines and Obesity. Potential Link to Metabolic Disorders and Chronic Complications

**DOI:** 10.3390/ijms21103570

**Published:** 2020-05-18

**Authors:** Katarzyna Zorena, Olga Jachimowicz-Duda, Daniel Ślęzak, Marlena Robakowska, Małgorzata Mrugacz

**Affiliations:** 1 Department of Immunobiology and Environment Microbiology, Medical University of Gdańsk, Dębinki 7, 80-211 Gdańsk, Poland; 2 Independent Public Specialized Health Care Center in Lębork, Department of Internal Diseases, Węgrzynowicza 13, 84-300 Lębork, Poland; oduda@interia.pl; 3 Department of Emergency Medicine, Faculty of Health Sciences, Medical University of Gdańsk, Smoluchowskiego 17, 80-214 Gdańsk, Poland; daniel.slezak@gumed.edu.pl; 4 Department of Public Health & Social Medicine, Faculty of Health Sciences, Medical University of Gdańsk, Al. Zwycięctwa 42a, 80-210 Gdańsk, Poland; marlena.robakowska@gumed.edu.pl; 5 Department of Ophthalmology and Eye Rehabilitation, Medical University of Bialystok, Kilinskiego 1, 15-089 Białystok, Poland; malgorzata.mrugacz@umb.edu.pl

**Keywords:** obesity, adipokines, inflammation, metabolic disorders, diabetic foot, psoriasis

## Abstract

The World Health Organization (WHO) has recognized obesity as one of the top ten threats to human health. It is estimated that the number of obese and overweight people worldwide exceeds the number of those who are undernourished. Obesity is not only a state of abnormally increased adipose tissue in the body, but also of increased release of biologically active adipokines. Adipokines released into the circulating blood, due to their specific receptors on the surface of target cells, act as classic hormones affecting the metabolism of tissues and organs. What is more, adipokines and cytokines may decrease the insulin sensitivity of tissues and induce inflammation and development of chronic complications. Certainly, it can be stated that in an era of a global obesity pandemic, adipokines may gain more and more importance as regards their use in the diagnostic evaluation and treatment of diseases. An extensive search for materials on the role of white, brown and perivascular fatty tissue and obesity-related metabolic and chronic complications was conducted online using PubMed, the Cochrane database and Embase.

## 1. Obesity: Definition

According to the World Health Organization (WHO), obesity is defined as “abnormal or excessive fat accumulation that presents a risk to health” [1]. In contrast, the World Obesity Federation (WOF) declared obesity itself as a chronic, relapsing progressive disease [2]. In the International Classification of Diseases, Eleventh Revision (ICD-11) WHO, the stigmatizing ICD-10 diagnosis “obesity due to excess calories” (E66.0) was not perpetuated [3]. Obesity is diagnosed when the percentage of body fat is higher than 25% in men and 30% in women [1]. Obesity is also recognized when the body mass index (BMI) is higher than 30 kg/m^2^ or when body mass exceeds 120% of the ideal body mass calculated from Brock’s formula [4,5,6]. Nowadays, there are a variety of methods available to assess body mass. However, the most precise methods are used for research purposes only. These include magnetic resonance, electrical conductivity and electrical bioimpedance [7].

## 2. Epidemiology of Obesity

Obesity became a global health problem as early as at the end of the 20th century. Nowadays, a pandemic of obesity is recognized [8,9,10]. Availability of highly processed foods, which are very easy to handle or do not require any handling and—most importantly—are very cheap, contributes greatly to the continuous increase in the incidence of obesity. The data presented by authors from all over the world are alarming [9,10,11,12,13]. Authors have shown that excess body weight impacts the dynamic increase in the incidence of hypertension, type 2 diabetes (T2DM) and ischaemic heart disease, not only in adults but also in adolescents [14,15,16,17,18]. The WHO estimates that there are more than 1.6 billion people living all over the world with BMI > 25 kg/m², including 522 million subjects with BMI beyond the obesity threshold >30/kg/m^2^ [1]. The high prevalence of the above stated disorders can be considered not only in terms of a medical problem, but also from economic and social perspectives. The estimated rate of premature deaths in Europe caused directly by obesity is 10–13% [13,19,20,21]. In the United Kingdom (UK) the problem of obesity affects 68% of adults. Approximately 5% of the UK health budget is spent each year on the treatment of obesity complications. Globalization and universality, as well as the continuously increasing popularity of so called “fast food”, results in the homogeneous prevalence of obesity in Eastern and Western Europe and the United States of America, making obesity a global health concern [22,23,24]. The increase in the percentage of people with abnormal body weight is no longer only a problem of highly developed countries as it is also observed in developing countries [9,10]. This phenomenon can easily be associated with economic factors as highly processed food is cheap and easily available. The nutritional value of food is very often a secondary issue, while the most important criterion of food choice is the economic factor [25,26]. 

## 3. Adipose Tissue 

Adipose tissue belongs to the class of connective tissues and is composed of adipocytes, preadipocytes, fibroblasts, stromal cells and macrophages [15,27,28]. Functions of the adipose tissue in the body include energy storage, thermal insulation, depreciation of internal organs and immune and endocrine function [27,28,29]. Until the 1980s the endocrine function of adipose tissue was unknown, and the fat tissue was regarded as an inactive store of energy accumulated in the form of triglycerides [29,30]. In the body of an adult man there should be on average 15–20% of fat tissue, while in the body of a woman the corresponding value ranges from 20 to 25%. Adipose tissue can be divided into white, brown, beige/brite and pink adipose tissue [29,30]. From a physiological point of view, all four types of adipose cells have endocrine properties. White adipocytes secrete a number of adipokines that affect eating behaviour and metabolism. Brown/beige adipocytes also secrete hormones and growth factors. Pink adipocytes, besides milk components, also secrete leptin [27,29,30].

**Figure 1:**
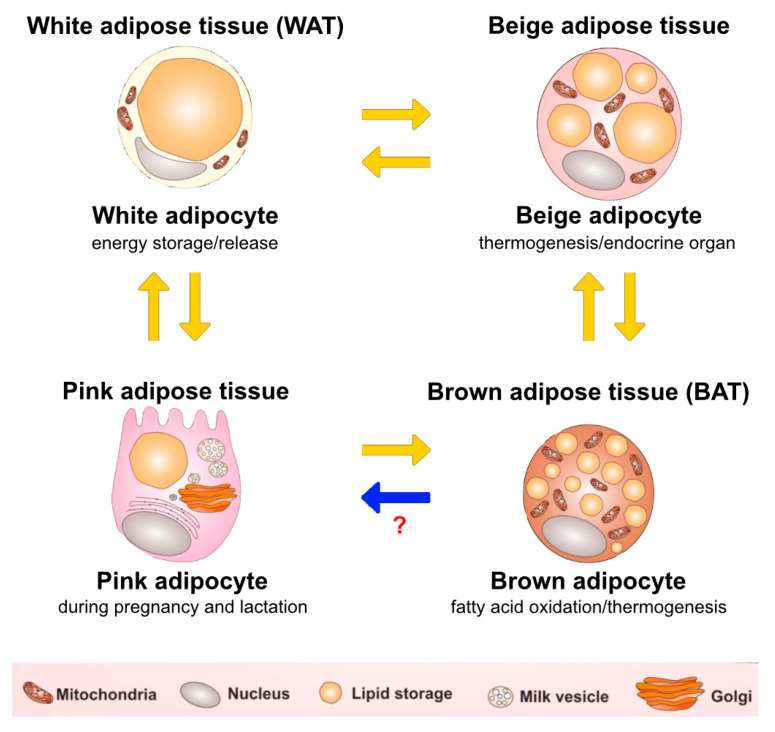
The conversion between adipocytes. Yellow arrows: white-brown-white adipocytes transdifferentiation, white-to-pink transdifferentiation, pink-to-brown transdifferentiation (🡆blue arrow: hypothesis which needs to be proved). Modified Figure 4 of [34], and Figure 2 of [35].

White adipocytes form white adipose tissue (WAT), which stores energy. Adipocytes of white adipose tissue are each filled with one large droplet of triglycerides, which makes the most of their cellular volume [29,30]. Both cellular organelles and cytoplasm are located peripherally. The white fat tissue is less vascularised and contains less extracellular matrix versus the brown fat tissue, which results from different functions of the two types of fat tissue [30,31]. WAT is divided into two regional and functional depots—vWAT and subcutaneous white adipose tissues (sWAT) [27,28,29]. vWAT is related to insulin resistance, inflammation, dyslipidemia, obesity and T2DM caused by the pathogenic expansion of WAT [18,27,28]. Conversely, sWAT is frequently associated with metabolic improvement and insulin sensitivity, as it contains brown-like cells known as beige adipocytes or inducible brown adipocytes that perform mitochondrial and thermogenic functions and burn fats [31,32,33]. The adipose organ has prominent plasticity ability. White adipocytes can be differentiated into brown-like adipocytes in WAT in a process called beiging [32,33,34]. Beige adipocytes are characterized by their multilocular lipid droplet morphology, high number of mitochondria and the expression of brown adipocyte genes [32,33]. Brown adipocytes raised in WAT are also identified as brite. These brite (brown-in-white) adipocytes are also known as beige, inducible brown or brown-like adipocytes [34,35]. The conversion between adipocytes is presented in Figure 1. Yellow arrows: white-brown-white adipocytes transdifferentiation, white-to-pink transdifferentiation, pink-to-brown transdifferentiation (blue arrow: hypothesis which needs to be proved). Modified Figure 4 of [34], and Figure 2 of [35].

Brown adipocytes are organized to form brown adipose tissue (BAT). Brown adipocytes are smaller than white adipocytes, and their cytoplasm contains several lipid droplets, a roundish nucleus and numerous, large, generally spherical mitochondria with laminar cristae [30,31]. These cells are also called multilocular adipocytes. Adipocytes of brown fat tissue are rich in uncoupling protein-1 (ucp-1), which is involved in accelerating heat production by uncoupling adenosine triphosphate (ATP) synthesis [32,33]. Energy obtained from fatty acid oxidation, instead of being stored in ATP, is dispersed in the form of heat. The brown fat tissue has an abundant extracellular matrix and rich vascularisation. It is found in fewer anatomical locations than white adipose tissue [31,34,35]. Molecular, immunohistochemical and electron microscopy studies from the last decades have revealed that the adipose organ is able to cooperate between WAT and BAT [29,31,33]. It has been found that the main function of the adipose organ is the division of nutrient-originated energy into two distinct pathways, i.e., WAT for metabolization and BAT for thermogenesis. In fact, during chronic exposure to cold, white adipocytes of WAT are transformed into BAT. This process is called browning or WAT to BAT conversion. On the other hand, during chronic positive energy balance, brown adipocytes of BAT are transformed into WAT. The process is called whitening [29,35].

The fourth type of adipocytes was presented as pink adipocytes [34,36]. Epithelial gland cells feature a characteristic abundance in cytoplasmic lipid drops, apical surface containing microvilli, big, round and centralized nucleus, rough endoplasmic reticulum (RER), Golgi apparatus and milk-containing granules [34,36]. Since the adipose organ is rendered pink during pregnancy and lactation, these epithelial adenocytes are called pink adipocytes. Immunohistochemical experiments using bitransgenic mice revealed that during murine pregnancy a so-called pinking, in other words, white-to-pink transdifferentiation or adipoepithelial conversion, is observed [36]. Once the lactation is over, the murine mammary gland is subject to rapid changes, which can be seen as early as within the first 24 h [29,34,36].

## 4. Adipokines and Metabolic Disorders 

The end of the 20th century saw increased interest in adipose tissue. This was caused by the rapidly growing prevalence of obesity all over the world [1]. A major breakthrough in the perception of adipose tissue as an endocrine organ was the discovery of the first adipokine, leptin [37,38]. Until now, a lot of substances secreted by adipose tissue have been discovered and described, and their metabolic effects and contribution to the pathogenesis of civilization-related diseases are being extensively studied [39,40,41,42]. It is interesting that both obese and lipodystrophic patients show similar clinical disorders: hypertriglyceridemia, insulin resistance and fatty liver [43,44,45]. The disorders lead to diabetes, hypertension, polycystic ovary syndrome (PCOS), coronary artery disease (CAD) and cancers [45,46,47,48,49,50]. 

**Figure 2:**
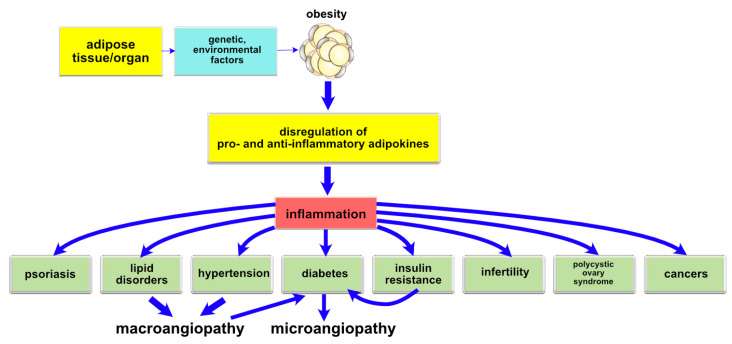
The disregulation pathway of adipokines potentially leading to metabolic disorders and chronic complications in the course of obesity.

The observations conducted have lead to the conclusion that adipose tissue in physiologically desirable quantities helps maintain body homeostasis. Substances secreted by adipose tissue control energy, lipid and carbohydrate metabolism in the body and can modulate immune system activity [51,52,53,54]. In an abnormal situation, such as obesity, adipose tissue does not perform its haemostatic functions anymore, which results in disregulation of the mechanisms involved in maintaining stability of the internal environment and activation of processes underlying the development of numerous metabolic disorders [54,55,56]. The disregulation pathway of adipokines potentially leading to metabolic disorders and chronic complications in the course of obesity is presented in Figure 2. The cancers have not been discussed, as this issue is beyond the subject covered in the manuscript.

It has been proven that inter-subject variability in the potential of adipose tissue expansion in order to store excess triglyceride quantities may affect metabolism disorders [57,58,59]. Hypertrophic adipocytes show pro-inflammatory potential and promote insulin resistance. Cells of this type synthesise high levels of pro-inflammatory cytokines, including interleukin 1 beta (IL1β), interleukin 6 (IL6) and tumour necrosis factor alpha (TNFα) [57,58]. Clinical studies of Pima Indians, who show high prevalence of obesity and T2DM, have confirmed that hypertrophic adipocytes favour the development of civilization-related diseases, including obesity-induced insulin resistance [60,61]. Small adipocytes show anti-inflammtory potential and result in increased glucose uptake by insulin sensitive tissues [60]. Moreover, it has been discovered that it is the size of the adipocytes rather than their number that correlates with the risk of nutrition-related disorders [61]. The imbalance between the energy intake with food and energy expenditures in metabolic processes and physical activity results in increased mass of adipose tissue. Adipose tissue in turn releases excessive amounts of adipokines that affect metabolism. Adipose tissue and the hypothalamus cross-talk enables appropriate interpretation of hunger and satiety signals [54,62]. There have been reports of considerable disorders concerning leptin and adiponectin functions. In obesity, hyperleptinemia can occur with accompanying leptin resistance in hypothalamic centres [63]. Adiponectin is the only adipokine that shows a negative correlation with visceral adipose tissue mass [59]. The anti-inflammatory effects of adiponectin include both the suppression of the production of pro-inflammatory factors (TNFα, IL6, CRP, etc.) and modulation of the expression of anti-inflammatory cytokines such as IL-10. On the other hand, pro-inflammatory factors suppress adiponectin production and regulate its levels [59]. 

## 5. Obesity, Cytokines and Inflammation 

Obesity is a disorder that favours the development of chronic inflammation. Excess adipose tissue and hypertrophic adipocytes lead to high levels of fibrynogen, CRP [64] and other acute phase proteins including (TNFα) [57], (IL6) [18,57] and interleukin 34 (IL34) [52,53] in the circulating blood. Increase in plasma pro-inflammatory cytokines induces vascular endothelial response. There is enhanced production of adhesion molecules, which, along with adipokine-induced chemokines, stimulate macrophage recruitment into adipose tissue. The resultant local inflammation promotes local insulin resistance [28,57]. A similar mechanism is seen peripherally, leading to systemic inflammation and subsequently to systemic insulin resistance [49,57]. A particular role is played by C-reactive protein (CRP), which is a sensitive and reproducible marker of inflammation [57,58,64]. It is synthesized in the liver in response to the proinflammatory cytokines (TNFα), IL1β and (IL6) [57,60]. CRP levels rapidly grow in the process of inflammation, which enables its use as a marker of inflammatory conditions. Due to the short elimination half-life of CRP, which is approximately 6 h, its levels depend mainly on its synthesis and rapidly fall after the causative factor disappears. Slightly increased CRP values, measured using a highly sensitive method, high-sensitivity C-reactive protein (hsCRP), indicate an inflammatory condition [64,65,66]. HsCRP is a key inflammatory marker associated with atherogenesis that is widely available, reliably standardized and precise. Low, moderate and high CAD risk correlate with values <1.0, from 1.0 to 3 and >3 mg/L, respectively, which has been observed in numerous population studies, including prospective studies [65,66]. Del Cañizo Gómez et al. [66] showed in their studies of 376 T2DM patients without diabetic complications that 4 years later diabetic microangiopathy developed in 95 patients (25.2%). Logistic regression analysis has shown that the main independent risk factors for the development of microangiopathy in T2DM patients were hsCRP > 3 mg/L and hypertension. The studies within the European Study on Cardiovascular Risk Prevention and Management in Usual Daily Practice (EURIKA study, 2014 ClinicalTrials.gov Identifier: NCT00882336) have indicated that CRP is actively involved in atherosclerosis [64]. The studies were conducted in 12 European countries with the aim of identifying coronary artery disease risk factors in 7565 subjects with at least one cardiovascular risk factor, including 5496 hypertensive subjects, 3288 obese subjects, 4372 dyslipidaemic subjects and 2027 diabetic subjects. The results showed that CRP levels were positively correlated with BMI and glycated haemoglobin and negatively correlated with high HDL cholesterol levels. 

Over 10 years ago it was observed that adipose tissue expressed interleukin 34 (IL34) [52,53,67]. High levels of IL34 were detected in the serum of obese patients compared to controls [67]. In addition, the authors observed a positive correlation between insulin-resistance-related metabolic parameters including BMI, systolic BP, fasting plasma insulin, HOMA-IR, serum leptin, hsCRP, VAT and SAT and higher levels of IL34 in VAT compared to SAT. Additionally, serum IL34 levels were shown to be high in patients with T2DM compared to controls, and operating characteristic curve analysis showed that IL-34 has more discriminatory power than CRP for the risk of diabetic complications [53]. 

Numerous data indicate that TNFα levels increase along with the severity of obesity [68,69,70,71]. In physiological conditions, exposure of fat tissue to growing levels of TNFα inhibits its further increase [68]. However, increased body mass sustained for a long time causes resistance to TNFα, which impairs the above-described mechanism and leads to further fat accumulation. Tumour necrosis factor is produced by adipose tissue and contributes to the pathogenesis of hypertension, especially obesity-associated hypertension. It has been shown that in a group of subjects with a BMI of 27 to 35 kg/m^2^ there is a statistically significant relationship between TNFα locus and obesity and hypertension loci [69]. Currently described potential TNFα paths are mainly associated with its indirect effect on insulin resistance stimulation. TNFα enhances lipolysis, thereby increasing serum free fatty acids and in this way favouring the development of insulin resistance [70]. Furthermore, via stimulation and activation of vascular adhesion molecules, TNFα favours atherogenesis [68,69,71]. Another mechanism involved in TNFα induction of insulin resistance in peripheral tissues is activation of nuclear factor-κB (NF-κB) and stimulation of the transcription of cytokines and adhesion molecules [72,73]. What is more, TNFα acts as a chemoattractant for monocytes and neutrophils and activates them similarly to macrophages. It enhances the cytotoxicity of monocytes and macrophages, at the same time being one of the cytotoxicity mediators. TNFα is one of the cytokines that induces breakdown of the blood-retinal barrier by opening tight junctions between retinal vascular endothelial cells and between retinal pigment epithelial cells [55,72]. Apart from its involvement in inflammatory processes, TNFα plays a significant role in neovascularisation and vasomotor response. TNFα secretion is markedly induced by hypoxemia and abnormally modified proteins that upregulate TNFα mRNA expression. Its numerous functions are mediated, among others, by its ability to induce synthesis of other cytokines functionally associated with TNFα, extracellular matrix proteins, modulation of monocyte and fibroblast chemotaxis, as well as impact on vascular adhesion molecule expression [73].

## 6. Obesity, Adipokines and Chronic Complications

Adipocytes and other cells of the adipose tissue are responsible for the production and secretion of numerous biologically active autocrine, paracrine and endocrine substances, including leptin, adiponectin, resistin, visfatin, chemerin, etc., which can lead to chronic complications. 

Leptin, a 16-kDa adipocyte-derived adipokine, is the product of the obesity (Ob) gene. Leptin activates macrophages/monocytes and natural killer cells and regulates the proliferation, phagocytosis, chemotaxis and oxygen radical release of neutrophils [74]. Leptin is produced mainly in mature cells of the WAT. Biosynthesis and secretion of leptin depends on the WAT mass and reflects the status of energy stores [74,75,76]. The main factors that affect the blood levels of leptin include fat tissue mass and adipocyte size. These measures show a positive correlation with leptin biosynthesis in fat tissue and its level in circulating blood [18,28]. Leptin is currently considered as a satiety hormone [76]. Leptin, released into circulating blood, is transported to the brain and bound to its receptors in the hypothalamus, where it causes repression of genes encoding neuropeptide Y (NPY) and induction of genes encoding proomiomelanocortin (POMC) and corticoliberin (CRH) [75,76]. This results in decreased appetite and reduced food intake with subsequent body fat reduction and increased energy expenditure, which finally leads to body mass decrease [76]. According to some authors, in the course of evolution, leptin appeared as a factor protecting against hunger or obesity at times of availability of excess food. Leptin is assumed to exert pleiotropic effects, affecting numerous metabolic paths [77]. Early studies of leptin levels and expression in human organs have shown that serum leptin levels increase along with increased body fat mass, which supports the hypothesis that white adipose tissue adipocytes are a rich source of this hormone [14,38]. Moreover, leptin has been shown to enhance insulin sensitivity in peripheral tissues and increase glucose uptake and oxidation in skeletal muscles [60]. Moreover, leptin affects thermogenesis through regulation of brown adipose tissue-specific mitochondrial proteins. It is involved not only in lipid and glucose metabolism and immune body response, but also in blood pressure control, blood coagulation and fertility [49,78,79]. 

Leptin is considered a potential marker of obesity-related complications [15,80,81]. Elevated leptin levels correspond to atherosclerosis [15,80] and neuropathy [81] but not diabetic retino- and nephropathy [81]. Csongrádi et al. [15] examined 154 obese individuals, including 98 suffering from atherosclerotic concomitant conditions, 56 free of atherosclerotic comorbidities, and 62 healthy controls. Adipokines were closely associated with markers of platelet hyperactivity, hypercoagulability, hypofibrinolysis and intima-media thickness (IMT). Furthermore, leptin (*p* = 0.0005), adiponectin (*p* = 0.019) and IL6 (*p* = 0.001) were independent predictors of IMT. The authors suggest that in obese subjects altered adipokine levels play a key role in common carotid atherosclerosis. In turn, Jung et al. [81] showed in their studies that serum leptin levels were not significantly different in patients with diabetic retino- and nephropathy, but were significantly higher in T2DM patients with neuropathy versus T2DM patients without neuropathy. 

Adiponectin is a 28 KDa protein with a similar structure to TNFα, collagen VIII and IV and complement factor C1q. In vitro studies have shown that adiponectin has antiatherogenic effects through inhibition of monocyte adhesion to endothelial cells and macrophage transformation into foam cells [82,83]. Moreover, adiponectin exerts its antiatherogenic effects via endothelial cell activation through decreased production of adhesion molecules and suppression of TNFα and transcription factor NFκB [84]. Adiponectin in blood vessel walls inhibits monocyte adhesion to endothelial cells as a result of downregulated expression of adhesion proteins and inhibits macrophage transformation into foam cells. Moreover, it inhibits smooth muscle cell proliferation, enhances nitrogen oxide synthesis and stimulates angiogenesis [15,59]. Numerous studies present adiponectin as an anti-inflammatory cytokine [83,84,85]. The anti-inflammatory effects of adiponectin are party due to the altered activity of TNFα. In vitro studies have shown that TNFα downregulates expression of the adiponectin gene via suppression of adiponectin-induced nuclear factor NFκB [84]. Studies in humans indicate reduced secretion of TNFα in adipose tissue in subjects with high adiponectin mRNA, whereas growing insulin resistance and increased body fat mass upregulate the expression of TNFα resulting in reduced adiponectin levels [83]. Adiponectin was also shown to directly increase IL10 production by macrophages and decrease production of proinflammatory cytokines TNFα and IL6 [85]. Adiponectin inhibits expression of adhesion molecules in vascular endothelial cells and production of cytokines in macrophages, thereby suppressing inflammatory processes occurring in the early phases of atherosclerosis and microangiopathy [86,87]. Increased serum adiponectin levels are believed to occur in response to vascular endothelial injury [88]. Adiponectin, reduced levels of which are associated with obesity, is also found in lower levels with incident hypertension [18]. On the other hand, authors have reported increased serum and urinary adiponectin levels in patients with diabetic nephropathy [87,89]. An association between adiponectin levels and degree of diabetic retinopathy has also been shown in patients with T2DM [81].

Resistin is an adipocytokine involved in the development of insulin resistance, which is reflected in the molecule’s name [89,90]. Resistin is a 12 kDa polypeptide that belongs to a unique family of cysteine-rich resistin-like molecules [89]. The main sources of resistin synthesis are peripheral blood inflammatory cells, monocytes and macrophages. The presence of resistin has also been shown in bone marrow, lungs, placenta, pancreatic islet cells and adipose tissue cells [90]. Some authors detected resistin in these cells as well as in inflammatory sites and peripheral blood [91,92,93]. Reilly et al. [91] investigated a possible association between resistin, inflammation, metabolic factors and atherosclerosis in healthy subjects and T2DM patients. Both groups showed increased resistin levels in females versus males and in T2DM patients versus healthy subjects. Resistin levels correlated with inflammatory markers, especially TNF-R2, in both studied populations. In patients with metabolic syndrome, resistin was a predictor of coronary artery atherosclerosis [93]. A study of 238 patients with T2DM demonstrated that serum levels of resistin were associated with the stage of diabetic retinopathy, nephropathy and neuropathy, regardless of age and gender, as well as BMI [94].

Visfatin is a 52 kDa protein product of the pancreatic beta cell growth factor (PEBF) gene, synthesised mostly by adipocytes and macrophages of adipose tissue and to a lesser extent by hepatocytes and neutrophils [95,96]. This adipokine is involved in the process of differentiation of preadipocytes to adipocytes and acts as a pre-beta lymphocyte colony stimulating factor. Moreover, it stimulates synthesis and storage of triacylglycerols in adipose tissue. Its production is regulated by numerous factors, with the most important role being played by TNFα [96]. It exerts its biological effects via the insulin receptor. It shows vasodilating effects (stimulates nitric oxide synthesis) but also pro-inflammatory actions by inducing the expression of adhesive molecules such as vascular cell adhesion molecule 1 (VCAM-1) and pro-inflammatory cytokines such as TNFα, IL1β and IL6 [97]. Moreover, visfatin stimulates endothelial cell proliferation, mediated by endothelial cell factor production, as well as smooth muscle cell growth [97,98]. However, studies on its role in the development of insulin resistance provided inconsistent results. In obese patients, increased visfatin levels, similarly to increased adiponectin levels, may play a protective role [99,100]. In a study by Kang et al, [100] on diabetic db/db mice it was shown that visfatin might have a protective effect in diabetic nephropathy without the hypoglycemic effect. In another study it was shown that, due to its relatively low levels, its effect on carbohydrate metabolism is negligible and glucose metabolism regulation ineffective [100]. On the other hand, there is a body of evidence to show adverse visfatin effects on insulin resistance [98,101]. Visfatin has pro-inflammatory properties mediated by leukocyte activation and stimulation of TNFα, IL6 and IL1β release, which impairs insulin signalling pathways. Studies by Chen et al. [102] have shown that visfatin is an independent risk factor for T2DM (OR 5.534; 95% CI 1.605–19.079; *p* = 0.007), and the risk of T2DM increases in each subsequent quartile. Increased visfatin levels in subjects with obesity/overweight, T2DM, metabolic syndrome and cardiovascular diseases have also been confirmed in a meta-analysis by Chang et al. [103]. 

Omentin and chemerin are adipokines that may modulate insulin action. They are also associated with obesity-induced insulin resistance. They are potential candidates to play a role in the pathogenesis of obesity and obesity-related diseases, including T2DM with or without vascular complications [104,105]. Omentin is a protein discovered in VAT but is found at lower concentrations in subcutaneous adipose tissue as well as in other tissues [106]. There are two omentin isoforms: omentin-1 and omentin-2. The first one is found first of all in circulating blood. Decreased levels of omentin-1 were detected in patients with impaired glucose tolerance and newly diagnosed, untreated T2DM [107]. Its levels are decreased in obese and overweight subjects and decrease when obese subjects lose weight [108,109]. Omentin inhibits osteoblast differentiation and vascular smooth muscle cell calcification. Decreased omentin levels in patients with visceral obesity have been suggested to cause the progression of artery calcification [107]. In a study by El -Messallamy et al. [108], decreased plasma levels of omentin-1 were detected in T2DM patients with CAD. Moreover, omentin levels were negatively correlated with obesity, hyperglycaemia, insulin resistance, inflammation and plasma chemerin levels. This reduction in omentin levels may result in decreased insulin-dependent glucose uptake in visceral and subcutaneous adipose tissues and other insulin-dependent tissues. IL-6 turned out to be an independent factor affecting omentin-1 levels [109].

Chemerin, similarly to omentin, potentiates insulin-dependent glucose uptake by adipocytes [106]. It is found in considerable amounts in adipose tissue, liver and immune cells and modulates the functions of these cells. Initially, it was recognized as a chemotactic factor for immune cells, including macrophages and dendritic cells [108,109]. Chemerin is also believed to be a link between obesity and inflammation. Its levels in humans are associated with numerous key elements of metabolic syndrome: BMI, triglycerides and arterial hypertension. Its levels are particularly high in very obese subjects, severe obesity [106,108,109]. It is secreted as an inactive precursor activated by serine proteases associated with cascades of coagulation, fibrinolysis and inflammation. In a study by El-Mesallamy et al. [108], chimerin levels were significantly increased in T2DM patients with concomitant obesity. Apart from this positive correlation with obesity, a positive correlation was seen with CRP and a negative correlation with HDL cholesterol and omentin.

## 7. Obesity, Adipokines and Psoriasis

Psoriasis is a chronic inflammatory multisystemic disease whose complex pathogenesis involves genetic, immune and environmental factors [88,110]. In the most up to date studies it was shown that there is a relation between mastocytes, T cells, neutrophils, inflammatory dendritic cells and hyperproliferative keratinocytes that lead to psoriatic lesions [111]. From the clinical point of view, the lesions are characterized by clear erythematous and scaly plaques, mainly located on the scalp, in the lumbosacral area, on the elbows, skin folds and knees [52]. Fernández-Armenteros et al. [112] conducted a comprehensive analysis of the relation between adipokines and psoriasis, highlighting that these bioactive products are directly associated with psoriasis and its co-morbidities, such as insulin-resistance, obesity, T2DM and cardiovascular diseases [112]. Adipocytes and inflammatory factors can contribute to dysregulation of the immune system and inflammation in psoriasis [113,114]. Bavoso et al. [113] detected significantly higher levels of leptin and lower adiponectin in patients with metabolic syndrome and psoriasis compared to the controls with metabolic syndrome. 

There is strong evidence to suggest that obesity is an independent risk factor for psoriasis [88]. Cytokines produced in the skin can in a direct way cause inflammation in fatty tissue, which results in obesity and vice versa—inflammatory mechanisms related to metabolic disturbances in the course of obesity can also directly affect inflammatory processes in psoriatic skin lesions. Several studies showed that white fatty tissue is the key place where pro-inflammatory adipokines such as leptin, adiponectin and resistin and standard cytokines such as IL6 and TNFα are formed [113,114]. Levels of leptin and resistin were higher in patients with psoriasis than in healthy people, and it strongly correlated with disease severity [113]. Furthermore, higher concentrations of TNFα and IL6 in the serum of obese patients with psoriasis are believed to be significant markers of psoriasis [114]. The results suggest that obesity, through pro-inflammatory pathways, is a factor predisposing patients to psoriasis and that obesity aggravates the psoriatic process. In accordance with the above, it has been pointed out that adipokines can be used as biomarkers to identify the stage of the disease and the risk associated with co-morbidities [113]. 

## 8. Obesity, Adipokines and Diabetic Foot 

The WHO defines diabetic foot syndrome as “ulceration of the foot (distally from the ankle and including the ankle) associated with neuropathy and different grades of ischemia and infection” [1]. Despite the efforts of numerous research teams, the pathogenesis of diabetic foot syndrome has not yet been fully elucidated. However, it is known now that diabetic foot is also associated with diabetic neuropathy, which can develop in the course of type 1 diabetes mellitus (T1DM) and T2DM [115]. The diverse clinical presentation and various onset times contribute to the fact that diabetic neuropathy is not always diagnosed at an early stage [116]. The most severe complication of diabetic neuropathy is the occurrence of slowly-healing ulcerations of the feet, which significantly deteriorate the quality of life of diabetic patients and consequently become a common reason for leg amputation and disability. The factors that increase the risk of diabetic foot syndrome with concomitant neuropathy mainly include ischemia caused by atheromatous lesions within the lower extremity arteries [117,118]. They often affect very small vessels of 2–4 mm in diameter, in which even a small narrowing results in severe limitation of blood flow and are usually multi-layer. An ischaemic foot is red but turns pale once it is lifted. It has shiny skin, no hair and its nails are thickened and deformed. On physical examination, there is usually no pulse palpable on the dorsal artery of the foot and the posterior tibial artery. The consequence of chronic ischaemia in the extremities is long-lasting wound healing and ineffective antibiotic therapy since antibiotics cannot penetrate the infected tissues [119,120]. Other significant risk factors for diabetic foot include immune disturbances and decreased immunity in diabetic patients. Consequently, foot ulcerations very quickly become infected by saprophytic and pathogenic bacteria, causing gangrene and necrosis [121,122,123]. The status of the immune system can be significant in several stages of the development of chronic wounds. Immune activation can precede ulceration in diabetic foot in the same way in which it precedes manifestation of T2DM and ischemic heart disease. Since pro- and anti-inflammatory processes are key in various stages of the wound healing process, it is possible that disturbances in the immune system disrupt homeostasis and wound healing and lead to characteristic chronic, non-healing wounds typical for diabetic foot syndrome. Recent studies showed lower levels of plasma adiponectin in patients with diabetic foot [124]. Moreover, the same authors observed a significant negative correlation between the level of plasma adiponectin and some cardiovascular risk factors, such as hypertension and dyslipidemia [123,124]. They analysed the volume of adipocytes and its relation to TNFα, IL6, adiponectin and hs-CRP levels. They showed that patients with diabetes and diabetic foot ulcerations at various stages had higher levels of IL6, hsCRP and TNFα and lower levels of plasma adiponectin compared to diabetic patients without foot ulcerations, regardless of coincident infections [124]. Other researchers detected a lower level of serum omentin in patients with T2DM and sensomotor polyneuropathy, irrespective of the applied risk factors of polyneuropathy [123,124]. The participation of selected pro- and anti-inflammatory adipokines in metabolic disorders and chronic complications in the course of obesity is presented Table 1.

## 9. Conclusions

Adipose tissue, the excess of which is found in obesity, is a source of numerous hormonally active substances, including adipokines. Adipokines, released into circulating blood, due to their specific receptors on the surface of target cells, act as classic hormones affecting the metabolism of tissues and organs. What is more, adipokines may decrease the insulin sensitivity of tissues and induce inflammation and the development of atherosclerosis, diabetes and psoriasis, as well as diabetic foot. Considering the complexity of chronic complications, it seems probable that it will be necessary to apply combined treatment, with pathways directed at various types of cells in various stages of the disease process. We hope that in the near future there will be new therapies proposed for patients with obesity. 

## Figures and Tables

**Table 1:** Participation of selected pro- and anti-inflammatory adipokines in metabolic disorders and chronic complications in the course of obesity.

Authors	Biochemical Factors	Concentration of Pro-Inflammatory and Anti-Inflammatory adipokines	Complications
Chandra et al. [18]	leptin adiponectin IL6	higher concentration of leptin and lower concentration of adiponectin	hypertension
Jachimowicz-Duda et al. [52]	IL34	higher concentration of IL34	lipid disorders, macroangiopathy, T2DM
Zorena et al. [53]	IL34	higher concentration of IL34	microangiophaty, macroangiopathy, T2DM
Malin et al. [54]	TNFα	higher concentration of TNFα	insulin resistance
Daniele et al. [57]	adiponectin TNFα, IL6, MCP1 osteopontin, fractalkine	higher concentration of adiponectin, TNFα, IL6, MCP1, osteopontin and fractalkine	hyperglycemia, insulin resistance, T2DM
Chang, et al. [67]	IL34	higher concentration of IL34	atherosclerosis, insulin resistance, blood pressure
Shivanna et al. [71]	TNFα	higher concentration of TNFα	blood pressure, insulin resistance, atherosclerosis
Elfassy et al. [78]	leptin	increased concentration of leptin	reduced fertility in obese men
Bou Nemer et al. [79]	leptin	increased concentration of leptin in follicular fluid of in obese women undergoing in vitro fertilization compared to follicular fluid from nonobese (normal weight and overweight) women	reduced fertility in obese women
Jung et al. [81]	leptin	increased concentration of leptin	neuropathy, T2DM
Alnaggar et al. [87]	adiponectin	increased serum and urinary of adiponectin	T2DM, nephropathy, hypertension
Reilly et al. [91]	resistin	increased concentration of resistin	inflammation, metabolic factors and atherosclerosis
Osawa et al. [94]	resistin	increased concentration of resistin	diabetic retinopathy, nephropathy and neuropathy
El-Mesallamy et al. [108]	chemerin omentin-1	increased concentration of serum chemerin decreased level of serum omentin-1	T2DM, ischaemic heart disease
Zhuang et al. [109]	chemerin	increased of chemerin concentration in healthy subjects but with first-degree relatives (FDRs) of T2DM patients	insulin resistance
Coimbra et al. [110]	TNFα, IL6, leptin resistin adiponectin	higher concentration of leptin, resistin, TNFα, IL6 and significantly lower concentration of adiponectin	psoriasis, overweight/obesity
Bavoso et al. [113]	leptin adiponectin	increased concentration of lepton and lower of adiponectin	disregulation of the immune system, inflammation, psoriasis, obesity
Tuttolomondo et al. [122]	adiponectin IL6	lower concentration of adiponectin and higher IL6	T2DM diabetic foot
Herder et al. [123]	omentin	lower concentration of omentin	diabetic sensorimotor polyneuropathy
Ahmad et al. [124]	adiponectin IL6 TNFα	lower concentration of adiponectin higher concentration of IL6 higher TNFα	T2DM diabetic foot retinopathy nephropathy neuropathy

Abbreviations: T2DM—type 2 diabetes mellitus, TNFα—tumor necrosis factor alpha; IL6—interleukin 6; IL34—interleukin 34.

## Abbreviations

WHO	World Health Organization
WOF	World Obesity Federation
ICD-11	International Classification of Diseases, Eleventh Revision
WAT	White adipose tissue
BAT	Brown adipose tissue
BMI	Body mass index
VAI	Visceral adiposity index
T2DM	Type 2 diabetes mellitus
T1DM	Type 1 diabetes mellitus
CAD	Coronary artery disease
IDF	International Diabetes Federation
PCOS	Polycystic ovary syndrome
CRP	C-reactive protein
hsCRP	high sensitivity C-reactive protein
L-LR	Leptin–leptin receptor
IL1β	Interleukin 1β
TNFα	Tumour necrosis factor alpha
IL6	Interleukin 6
IL34	Interleukin 34
NF-Κb	Nuclear factor-κB
UCP-1	Uncoupling protein-1
PDR	Proliferative diabetic retinopathy
